# Rituximab Therapy for Mucous Membrane Pemphigoid: A Retrospective Monocentric Study With Long-Term Follow-Up in 109 Patients

**DOI:** 10.3389/fimmu.2022.915205

**Published:** 2022-06-30

**Authors:** Gérôme Bohelay, Marina Alexandre, Christelle Le Roux-Villet, Ishaï Sitbon, Serge Doan, Isaac Soued, Jason Shourick, Laurie Rousset, Benoît Mellottee, Michel Heller, Nicole Lièvre, Coralie Zumelzu, Florence Morin, Sabine Grootenboer-Mignot, Eric Gabison, Frédéric Caux, Catherine Prost-Squarcioni, Philippe Musette

**Affiliations:** ^1^ Department of Dermatology and Referral Center for Autoimmune Bullous Diseases (MALIBUL), Avicenne Hospital, Hôpitaux Universitaires de Paris Seine-Saint-Denis, AP-HP, Université Sorbonne Paris Nord, Bobigny, France; ^2^ Inserm UMR 1125 Li2P, UFR SMBH Léonard de Vinci, Université Sorbonne Paris Nord (USPN), Bobigny, France; ^3^ Department of Ophthalmology, Bichat University Hospital, AP-HP, Université de Paris, Paris, France; ^4^ Department of ENT and Referral Center for Autoimmune Bullous Diseases (MALIBUL), Avicenne Hospital, Hôpitaux Universitaires de Paris Seine-Saint-Denis, AP-HP, Bobigny, France; ^5^ Department of Epidemiology, Clinical Epidemiology and Public Health, UMR 1027 INSERM-University of Toulouse III, Toulouse University Hospital (CHU), Toulouse, France; ^6^ Department of Histology, UFR SMBH Léonard de Vinci, Université Sorbonne Paris Nord (USPN), Bobigny, France; ^7^ Department of Immunology and Referral Center for Autoimmune Bullous Diseases (MALIBUL), Saint Louis University Hospital, AP-HP, Université de Paris, Paris, France; ^8^ Department of Immunology and Referral Center for Autoimmune Bullous Diseases (MALIBUL), Bichat University Hospital, AP-HP, Université de Paris, Paris, France; ^9^ Department of Pathology, Avicenne University Hospital, Hôpitaux Universitaires de Paris Seine-Saint-Denis (HUPSSD), AP-HP, Université Sorbonne Paris Nord (USPN), Bobigny, France

**Keywords:** rituximab, mucous membrane pemphigoid, fibrotic conjunctivitis, epidermolysis bullosa acquisita, linear bullous IgA dermatosis, autoimmune bullous diseases

## Abstract

Mucous membrane pemphigoid (MMP) is a heterogeneous group of rare, chronic, subepithelial autoimmune blistering diseases (AIBDs) with predominant involvement of mucous membranes that can be sight-threatening and life-threatening. Rituximab (RTX) has demonstrated its efficacy in severe MMP refractory to conventional immunosuppressants in small series that differed in RTX scheme, concomitant therapies, and outcome definitions. In a meta-analysis involving 112 patients with MMP treated with RTX, complete remission (CR) was reported in 70.5% of cases. Herein, we report the largest retrospective monocentric study on RTX efficacy in a series of 109 severe and/or refractory patients with MMP treated with RTX with a median follow-up period of 51.4 months. RTX was administered in association with immunomodulatory drugs (dapsone, salazopyrine) without any other systemic immunosuppressant in 104 patients. The RTX schedule comprised two injections (1 g, 2 weeks apart), repeated every 6 months until CR or failure, with a unique consolidation injection (1 g) after CR. The median survival times to disease control and to CR were 7.1 months and 12.2 months, respectively. The median number of RTX cycles required to achieve CR in 85.3% of patients was two. The larynx was the lesional site that took the longest time to achieve disease control. One year after RTX weaning, CR off RTX was obtained in 68.7% of cases. CR off RTX with only minimum doses of immunomodulatory drugs was achieved in 22.0% of patients. Further, 10.1% of patients were partial responders and 4.6% were non-responders to RTX. Relapse occurred in 38.7% of cases, of whom 91.7% had achieved CR again at the last follow-up. In MMP, CR was achieved in a longer time and after more rituximab cycles than in pemphigus, especially for patients with MMP with anti-type VII collagen reactivity. RTX with concomitant immunomodulatory drugs was not responsible for an unusual proportion of adverse events. This large study confirms that RTX is an effective therapy in patients with severe and/or refractory MMP, corroborating previous findings regarding the effects of RTX on AIBDs such as pemphigus.

## Introduction

Mucous membrane pemphigoid (MMP) comprises a heterogeneous group of rare, chronic, autoimmune subepithelial blistering diseases responsible for blistering and erosions with predominant involvement of mucous membranes and a tendency of scarring (1–3). MMP diagnosis relies on clinical examination, histological examination, and the identification of immune deposits along the basement membrane zone on direct immunofluorescence (DIF) or direct immunoelectron microscopy (DIEM) (2). Serum immunological analyses may identify auto-antibodies directed against several basement membrane antigens such as BP180, BP230, laminin-332, *α*6*β*4-integrin, and collagen VII (2). MMP primarily affects the oral and conjunctival mucosa but may involve all malpighian mucous membranes. Patients with mild disease, involving only the oral mucosa and/or skin, might achieve complete remission (CR) with topical corticosteroids or with immunomodulatory drugs, such as dapsone, sulfasalazine, and tetracycline (2, 4–6). However, in severe cases with the involvement of multiple sites or isolated ocular, or laryngo-tracheal/esophageal mucous membrane involvement, a more aggressive first-line approach is usually employed to prevent the consequences of mucous membrane inflammation and scaring that might lead to irreversible sequelae or death. In such patients, conventional immunosuppressants (ISAs) such as cyclophosphamide or mofetil mycophenolate, alone or in association with corticosteroids, have been shown to have good efficacy and are usually rapidly started (2, 7–9). Nevertheless, the latter might be insufficient or contra-indicated. In studies with small series, biologic therapies such as off-label rituximab (RTX) have been shown to be useful in achieving CR in MMP cases. In 2011, we published a series of 25 patients with severe and/or refractory MMP treated with RTX, of whom 88% achieved CR after one or two cycles of RTX (10). As per recent European guidelines, RTX is indicated in association with dapsone for the treatment of MMP refractory to ISAs (2). Recently, a systematic review investigated the literature regarding MMP treated with biologics and concluded that 70.5% of 112 patients treated with heterogenous RTX regimens achieved CR, with a 35.7% recurrence rate during a mean follow-up period of 1.9 years (11). Since MMP is a rare disease, controlled studies are difficult to conduct and retrospective studies of well-characterized patients in real clinical settings with long term follow-up are of significant interest. Herein, we provided the outcomes of 109 patients with MMP treated at our center with the same off-label RTX protocol during a 10-year period to investigate the efficacy of RTX in MMP according to outcome definitions outlined in the 2015 expert consensus statement on MMP (12). Statistical analyses were performed to identify the factors associated with CR, and relapse.

## Methods

This single center, retrospective study was conducted on patients followed-up between 2009 and 2021 at Avicenne Hospital (Assistance Publique-Hôpitaux de Paris, Bobigny, France), using the computer database (eDBAI) of the referral center for AIBDs. We obtained local institutional review board approval to conduct this study (#CLEA-2022-236).

### Patient Selection

All patients with a diagnosis of MMP who received RTX at our center between 2009 and 2019 were identified by a computer search in the eDBAI database and were screened for inclusion. This inclusion period avoided the screening of patients reported in our previous study (10). To accurately evaluate RTX efficacy, we excluded MMP cases having received RTX for a surgical procedure while being in CR, those with less than 6 months of follow-up after baseline, or those with concomitant initiation of RTX and another biologic therapy (e.g., intravenous immunoglobulins, omalizumab) during the same period.

### Standard Assessment of AIBD in our Referral Center

**Figure 1:**
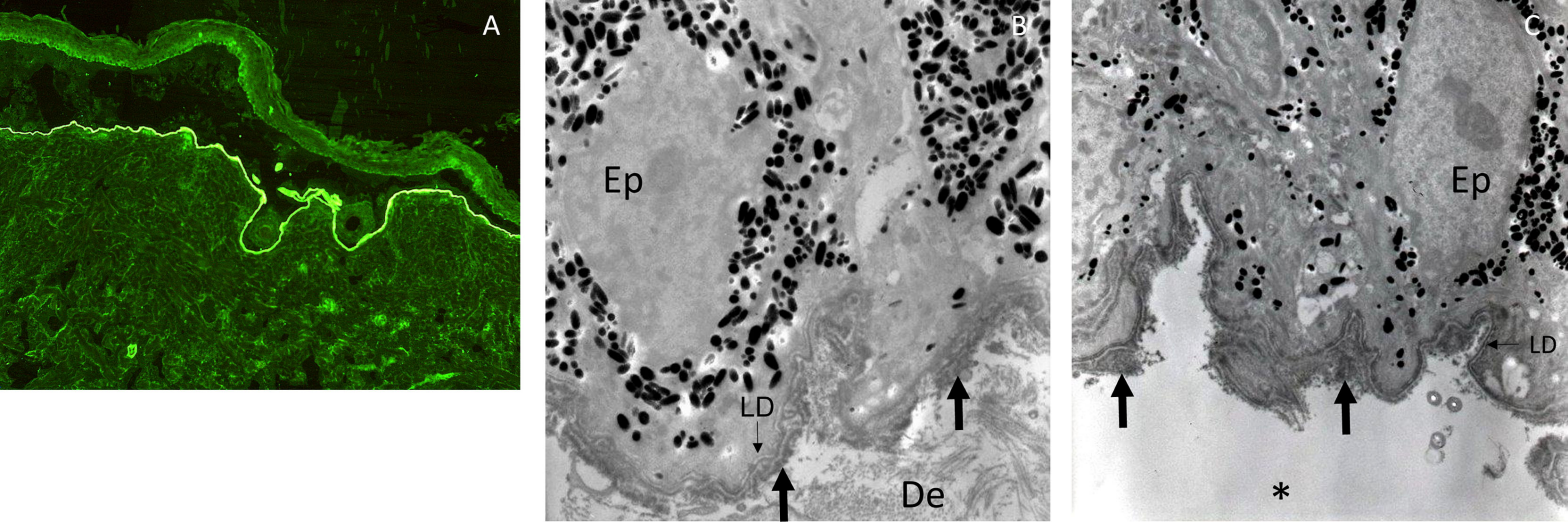
Immune deposits in indirect immunofluorescence microscopy on salt-split skin and in direct immunoelectron microscopy in a patient with MMP with anti-type VII collagen reactivity. Indirect immunofluorescence microscopy on primate salt-split skin showing a labeling of the floor of the cleavage by the serum of a patient with MMP with anti-type VII collagen reactivity **(A)**. Direct immunoelectron microscopy in a patient with MMP with anti-type VII collagen reactivity showing thick IgG **(B)** and C3 **(C)** deposits (large arrows) in the anchoring fibril zone below the lamina densa (LD, thin arrows) and split (asterisk) below them (Ep, epidermis; De, dermis).

All patient information was systematically recorded and stored in a computerized medical chart standardized for AIBDs after obtaining written informed consent from the patients. The definite diagnosis of subepithelial AIBD and its type relied on a multidisciplinary clinical assessment recording past medical history; cutaneous and mucous membrane lesions; as well as histological and immunological tests, as recommended (2, 3) and previously reported (13). The methods used included direct immunofluorescence (DIF), indirect immunofluorescence (IIF) on rat and/or monkey esophagus and primate salt-split skin, direct immunoelectron microscopy (DIEM), serum anti-BP180-NC16A and anti-BP230 IgG using commercial enzyme-linked immunosorbent assays (ELISAs), serum anti-collagen VII IgG using commercial and/or in-house ELISA (14), and IgG immunoblotting performed with human amniotic membrane extract (15). A multidisciplinary clinical assessment was systematically performed including, at first visit and during follow-up, dermatologists, stomatologists, ophthalmologists, and otorhinolaryngologists from the referral center. The scoring of the fibrotic component of ocular involvement used the stages described by Foster et al. (3, 16). In cases of MMP with isolated chronic fibrotic conjunctivitis, serum immunological analyses and DIF of another uninvolved site were frequently negative, and conjunctival biopsy for DIEM analysis may be postponed or not carried out considering the risk of worsening the sight-threatening ocular scarring in case of diffuse activity. In such cases, chronic conjunctivitis with limbitis may be regarded as a distinctive sign of ocular MMP (3). Thus, in evocative cases of ocular MMP without DIF or DIEM positivity, MMP diagnosis relied on clinical exclusion of alternative diagnoses (2). MMP with anti-type VII collagen reactivity were defined according to DIEM (electron-dense immune deposits on the anchoring fibrils, **Figure 1**) results and/or presence of circulating anti-type VII collagen antibodies in accordance with the 2018 consensus on epidermolysis bullosa acquisita (EBA) (17).

### RTX Administration Schedule in Patients With MMP

A collegial decision of the department of dermatology to begin RTX administration as an off-label therapy was taken considering our previous experience in the field with patients with MMP in therapeutic impasse and/or bearing comorbidities that did not allow the use of other conventional ISAs (2, 9, 10). In patients with a history of cancer in remission, the agreement of the referring oncologist was required to start RTX therapy. The RTX schedule comprised a first cycle at baseline (two infusions of 1 g, 2 weeks apart). Conventional ISAs (*e.g.*, cyclophosphamide, mofetil mycophenolate) were stopped before RTX therapy which was used in combination with immunomodulatory therapies (*e.g.*, dapsone, sulfasalazine, tetracyclines). If patients already received topical or systemic corticosteroids, a stable dose was maintained until disease control (DC). Some patients with MMP with severe disease and without clinical improvement at the 3-months follow-up from baseline received a second cycle of RTX (two infusions of 1 g, 2 weeks apart) at this point. From the sixth month from baseline, additional cycles (two infusions of 1 g, 2 weeks apart) were repeated every 6 months until CR. After reaching CR, patients with MMP had a consolidation cycle of RTX (one infusion of 1 g, 6 months apart from the last cycle) before RTX cessation. In relapsing patients after RTX cessation, RTX was resumed according to the same schedule. For patients exhibiting partial remission or chronic relapse, RTX infusions were continued every 6 months with one or two infusions of 1 g, 2 weeks apart, on an individual basis. In case of RTX failure (see below), RTX administration was stopped, and an alternative therapy was commenced. The management of therapeutic de-escalation after reaching CR comprised the maintenance of immunomodulatory drugs at the same dosage until CR off RTX, before their progressive tapering to achieve CR off RTX with minimal therapy.

### Outcome Definitions

Outcomes were defined according to the 2015 consensus statement for MMP (12). The baseline was defined as the day that patients with MMP were administered the first RTX injection. DC was defined as the absence of new lesions with only established lesions in healing. DC was reported for each site individually and for all sites involved. CR was defined as the absence of new non-transient or healing of established lesions for 2 months in all sites involved; CR on RTX and CR off RTX were defined according to the time from the last RTX infusion in CR patients; those still in CR 1 year after the last RTX infusion were classified as CR off RTX. CR off RTX with minimal therapy was defined as CR off RTX with minimal doses of concomitant immunomodulatory drugs as defined in the 2015 consensus statement (*e.g.*, dapsone ≤1.0 mg/kg/d, salazopyrin 1 g/d, doxycycline 100 mg/d, colchicine 0.5 mg/d). Partial response was defined as the halving of the activity part of the MMP disease area severity index (MMPDAI) score in comparison with baseline. Patients who did not reach partial response were considered as non-responders. Patients with absence of improvement after the first two RTX cycles or with insufficient response subsequently (non-responders or partial responders) with persistence of mucous membrane involvement at high risk of complications such as ocular, laryngo-tracheal, and esophageal involvement were considered as RTX failure. Time to DC and time to CR were defined from baseline to the date of the first visit presenting with DC or CR, respectively. Relapse was defined as non-transient lesion occurrence. The endpoint of the follow-up was defined as the last visit or the date on which RTX was replaced by another therapy for patients with RTX failure.

### Collected Data

The data collected included baseline information, sex, results of diagnostic investigations, age at diagnosis, time duration between first symptoms and diagnosis, clinical involvement at diagnosis, and treatment lines before RTX.

At baseline, gender, age, time duration between first symptoms/diagnosis and baseline, indication for RTX therapy, concomitant treatments received from baseline in association with RTX, data on clinical involvement, and MMPDAI (activity score) were collected. For topical corticosteroids, only the skin application of high-potency topical corticosteroids (betamethasone dipropionate or propionate clobetasol ≥10 g/d) was recorded.

During follow-up, the response to RTX (no response, partial response, CR, CR off RTX, CR off RTX with minimal therapy), time to achieve DC for each site involved and for the whole sites, time to achieve CR, number of RTX cycles and injections to achieve DC and CR, date and treatment of relapses, adverse events, number of RTX cycles and injections at last follow-up, concomitant treatments at last follow-up, and follow-up duration were collected. Lymphopenia was defined as a lymphocyte blood count <1.0 G/L. Neutropenia was defined as a neutrophil blood count < 0.5 G/L. Severe adverse events were defined as grade 3 adverse events according the common terminology criteria for adverse events (CTCAE v5.0) (18).

### Statistical Analysis

Descriptive and comparative analyses were computed with StatView software (v5.0, SAS Institute Inc). Quantitative variables were expressed as medians and interquartile range or extreme values, as indicated, according to normality assessed by the Shapiro-Wilk test. Time to DC, time to CR, and time to relapse were determined with Kaplan-Meier survival curves and were expressed as median survival times and standard deviations. Qualitative variables were presented as numbers and proportions. Adverse events were expressed as number, proportions, and incidence per 100 person-year, which was calculated based on the follow-up duration from baseline, except for coronavirus disease 2019 (COVID-19)-related adverse events that only included the patients followed-up during the COVID-19 pandemic.

To identify factors influencing the CR, patients in CR and those without CR were compared at the 8-month follow-up and included only patients with 8 months of follow-up after baseline. CR was studied at the 8-month follow-up because, considering the definition of CR given above, it corresponded to complete healing without new lesions from the 6-month follow-up and afforded sufficient numbers of patients in groups for statistical analyses. Analyses aimed at identifying factors influencing the relapse included only patients having reached CR and having ≥12 months of follow-up after the CR date. For quantitative variables, univariate comparisons between the different subgroups of patients with MMP were performed using Mann–Whitney tests, but for time to CR, log-rank tests were used. For qualitative variables, univariate comparisons were performed using Pearson’s χ2 tests, with or without Yate’s continuity correction, or Fisher’s exact tests, as appropriate, according to the size of the sample. The factors associated with response to RTX were identified by univariate and backward stepwise multivariate logistic-regression analyses, with their respective significance levels set at <0.20 and <0.05.

Besides, to assess factors influencing the CR, time to CR determined with Kaplan-Meier survival curves were studied for clinical and immunological parameters. For these univariate comparisons, log-rank tests were used. Prism^®^ software (GraphPad Software Inc., San Diego, CA, USA) was used to perform the figures of Kaplan-Meier survival curves.

## Results

### Study Population Before RTX Therapy

**Figure 2:**
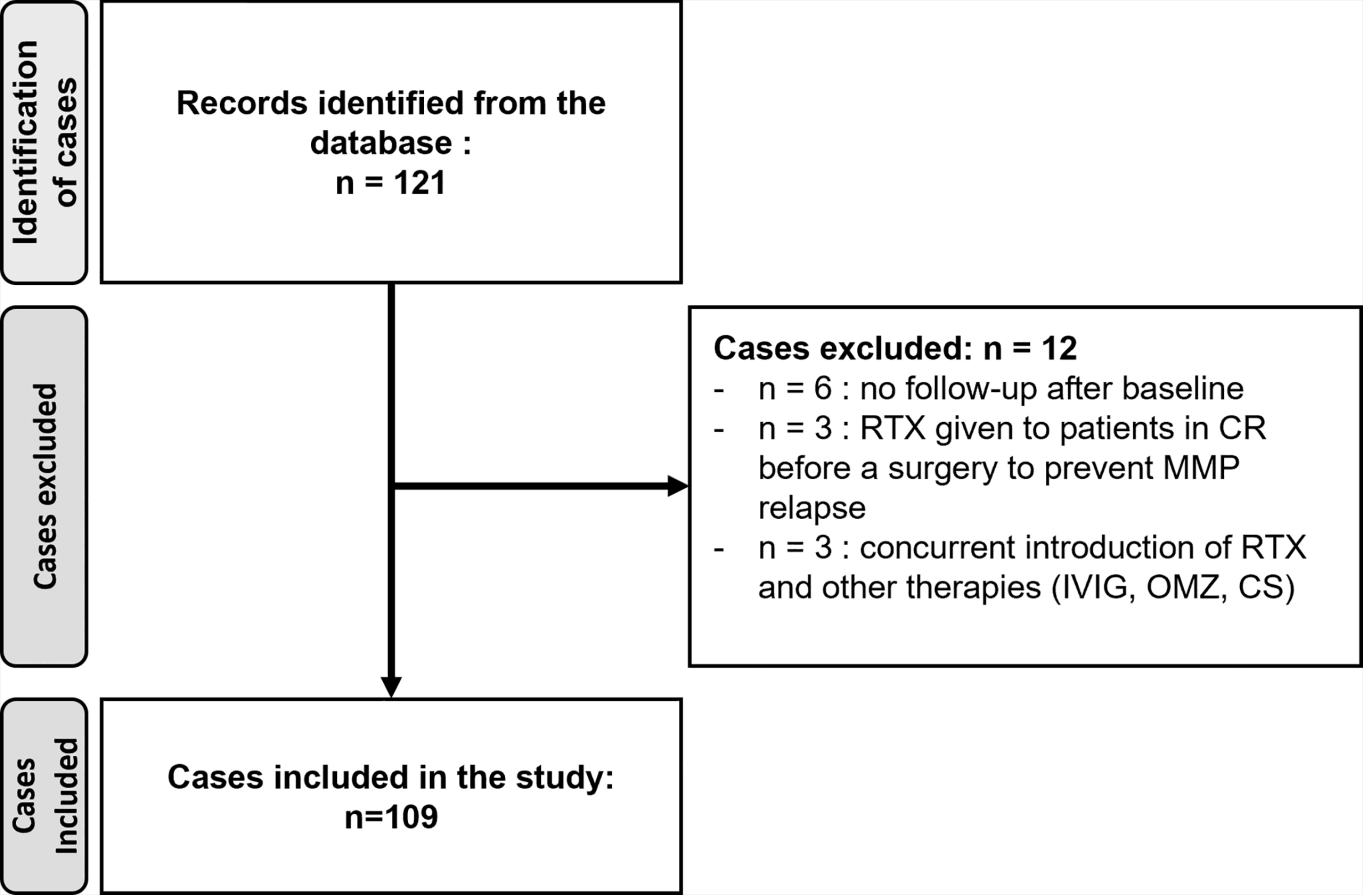
Study flow chart.

We identified 121 patients with MMP who received RTX during the study period. Twelve were not included in the study based on the exclusion criteria (**Figure 2**). The study cohort included 109 patients (**Table 1**). There was a slight majority of females (51.4%). The median age at MMP diagnosis was 69.7 years and the median time between first symptoms and diagnosis was 15.9 months (range: 0–475 months). The diagnosis had been confirmed by the identification of linear immunoglobulins or complement deposits on the basement membrane zone on DIF and/or DIEM in 93 patients (85.3%). Specifically, 81 patients (74.3%) had immune deposits on DIF, and 76 patients (69.7%) had immune deposits in DIEM of whom 12 were negative on DIF (11.0%). IIF was positive in 36 patients (33.0%) and circulating antibodies were found in 52 patients (47.7%) in ELISA or immunoblotting. The antibodies against specific basement membrane antigens found in ELISAs or immunoblotting were antibodies to BP180 (29.4%, n = 32), collagen VII (9.2%, n = 10), BP230 (6.4%, n = 7), laminin 332 (5.5%, n = 6), LAD-1 (3.7%, n = 4), and α6 subunit of α6β4 integrin (1.8%, n = 2). Three patients had a negative result for anti-BP180-NC16A ELISA but had a 180-kDa band by immunoblotting. Ten patients (9.2%) had circulating antibodies to several antigens of the basement membrane zone. Nine patients were diagnosed as MMP based on clinical examination, after alternative diagnoses had been ruled out, in the absence of immune deposits in DIF (n = 6) or in DIEM (n =3) and without circulating antibodies in ELISA. In these nine cases, the MMP involved a unique mucous membrane: conjunctival in eight and tracheal in one.

**Table 1:** Mucous membrane pemphigoid characteristics at diagnosis.

Characteristics	All MMPN = 109	
Female gender, N (%)	56	(51.4)
Age at diagnosis (years), median (IQR)	69.7	(21.6)
Delay between symptoms and diagnosis (months), median (IQR)	15.9	(30.4)
Immune deposits on BMZ		
Immune deposits in DIF* IgG, N (%) IgA, N (%) IgM, N (%) C3, N (%)	64 29 2 60	(66.0) (29.9) (2.1) (61.9)
Immune deposits in DIEM or DIF^†^ IgG, N (%) IgA, N (%) C3, N (%)	78 39 77	(77.2) (38.6) (76.2)
Anti-BP180 antibodies, N (%)	32	(29.4)
Involvement at diagnosis Muco-cutaneous, N (%) Mucous only, N (%)	53 56	(48.6) (51.4)
MMP with anti-type VII collagen reactivity, N (%)	12	(11.0)
Sites affected at diagnosis Ocular, N (%)	65	(59.6)
Nose and throat, N (%)	74	(67.9)
Larynx/Trachea, N (%)	54	(49.5)
Esophageal, N (%)	9	(8.3)
Genital, N (%)	34	(31.2)
Anal, N (%)	20	(18.3)
Buccal, N (%)	71	(65.1)
Cutaneous, N (%)	53	(48.6)
Number of sites involved, median (IQR)	3	(2.0)
≥3 sites involved, N (%)	73	(67.0)
Severe MMP, N (%)	104	(95.4)
Prior failed treatments		
Immunomodulatory drugs^¶^, N (%)	88	(80.7)
DDS/salazopyrine, N (%)	74	(67.9)
ISAs, N (%)	72	(66.1)
Cyclophosphamide, N (%)	55	(50.5)
Prednisone, N (%)	12	(11.0)
MMF, N (%)	7	(6.4)
Cyclosporine, N (%)	4	(3.7)
IVIG, N (%)	4	(3.7)
Azathioprine, N (%)	2	(1.8)
MTX, N (%)	2	(1.8)
RTX^‡^, N (%)	1	(0.9)
Previously had a period of CR before baseline, N (%)	10	(9.2)

MMP, mucous membrane pemphigoid; IQR, interquartile range; BMZ, basement membrane zone; DIF, direct immunofluorescence; DIEM, direct immuno electron microscopy; DDS, dapsone; ISAs: immunosuppressive agents; MMF: mofetil mycophenolate; IVIG, intravenous immunoglobulins; MTX: methotrexate; RTX, rituximab. *based on 97 cases who had DIF; ^†^based on 101 cases who had DIF or DIEM; ^¶^ i.e., DDS, sulfasalazine, doxycycline or equivalent, hydroxychloroquine, acitretin or colchicine; ^‡^this patient had had one RTX cycle 5 years before baseline in another center.

Strictly mucosal involvement was observed in 51.4% of the 109 patients, and mucocutaneous involvement was observed in 48.6%. Severe MMP accounted for 95.4% of cases with ≥3 mucosal sites involved in 67.0% of patients and/or a mucosal involvement at high risk of complication; ocular conjunctivae, larynx, or esophagus were involved in 59.6%, 49.5%, and 8.3% of cases, respectively. A previous period of CR before baseline was only reported in 9.2% of cases; 80.7% of patients had undergone non-immunosuppressive treatments, such as dapsone and/or salazopyrine (67.9%), and 66.1% had been administered one or more conventional ISAs, notably cyclophosphamide (50.5%).

### Study Population at Baseline

RTX therapy was commenced in patients with MMP with a median age of 70.5 years (range: 16–93 years), after a median time of 35.8 months between the first symptoms and baseline (range: 1.3–486.0 months) and a median time of 7.4 months between the diagnosis and baseline (range: 0.3–280.7 months) (**Table 2**). RTX therapy was commenced in patients with active MMP having a median MMPDAI score of 10.9 (range: 0–52) with a median number of two sites involved (range: 1–6 sites); ≥3 sites were involved at baseline in 40.4% of cases. The most common mucosal sites involved were the mouth (61.5%), conjunctivae (51.4%), and larynx/trachea (39.4%); laryngeal involvement is not included in MMPDAI explaining that some patients had a value of zero for the MMPDAI score. All 56 patients with conjunctival involvement had severe involvement with Foster’s stage ≥IIC, of whom 49 patients (87.5%) had Foster’s stage ≥III, for at least one eye. Patients with MMP had an active disease refractory to immunomodulatory drugs (*i.e.*, dapsone, salazopyrine, colchicine, acitretin, and doxycycline) and conventional ISAs in 82.6% and 66.1% of cases, respectively. The other factors that led to RTX use were treatment side-effects, contraindication to conventional ISAs, and non-compliance to treatments (28.4%, 22.9%, and 5.5%, respectively). At baseline, the concomitant therapies used with RTX comprised immunomodulatory drugs (78.0%), systemic corticosteroids (4.6%), and skin application of topical corticosteroids (15.6%). No patient had other conventional immunosuppressants at baseline.

**Table 2:** Patient characteristics at baseline.

Characteristics	All MMPN = 109	
Age at baseline (years), median (IQR)	70.5	(20.0)
Time duration (months) between first symptoms and baseline, median (IQR)	35.8	(60.6)
Time duration (months) between diagnosis and baseline, median (IQR)	7.4	(13.2)
Baseline Involvement		
MMPDAI activity score, median (IQR)	10.9	(13.2)
Number of sites involved, median (IQR)	2	(1–6)
≥1 site involved, N (%)	70	(64.2)
≥2 sites involved, N (%)	44	(40.4)
Ocular, N (%)	56	(51.4)
Larynx and/or trachea, N( %)	43	(39.4)
Buccal, N (%)	67	(61.5)
Skin, N (%)	30	(27.5)
Genital, N (%)	12	(11.0)
Anal, N (%)	12	(11.0)
Esophagus, N (%)	7	(6.4)
RTX indication		
Refractory/contraindication to immunomodulatory drug^¶^, N (%)	89	(81.7)
Refractory to ISAs, N (%)	71	(65.1)
Time duration of ISAs treatment, median (IQR)	5.0	(5.7)
Contraindication to conventional ISA, N (%)	25	(22.9)
Treatment side effects, N (%)	31	(28.4)
Non-compliance with treatments, N (%)	6	(5.5)
Concomitant therapies at baseline		
Immunomodulatory drugs^¶^, N (%)	85	(78.0)
DDS and/or salazopyrine, N (%)	72	(66.1)
Topical corticosteroids^‡^, N (%)	17	(15.6)
Systemic corticosteroids, N (%)	5	(4.6)

MMP, mucous membrane pemphigoid; IQR, interquartile range; MMPDAI, mucous membrane pemphigoid disease activity index; ISA, immunosuppressive agents; DDS, dapsone. ^¶^i.e., DDS, Sulfasalazine, doxycycline or equivalent, hydroxychloroquine, acitretin or colchicine; ^‡^cutaneous application of more than 10g/d of high potent topical corticosteroids.

### Clinical Response After RTX Adjunction to Concomitant Treatments and Outcome

During the median follow-up time of 51.4 months (range: 1.2–132.8 months), patients with MMP received a median value of four cycles of RTX corresponding to six RTX infusions (**Table 3**). Most patients received ≥2 RTX cycles (89.0%). Within 1 year after baseline, patients with MMP received a median value of two RTX cycles (range: 1–4 cycles). The median time between the first two cycles of RTX was 6.1 months, but 24 patients (22.0%) underwent a second cycle before the fourth month from baseline.

**Table 3:** Outcome of rituximab therapy in patients with mucous membrane pemphigoid.

Characteristics	All MMPN = 109	
Follow-up duration (months), median (range)	51.4	(1.2–132.8)
Total number of RTX cycles, median (IQR)	4.0	(4.0)
Total number of RTX injections, median (IQR)	6.0	(6.0)
Number of RTX cycles within 1st year after baseline, med (IQR)	2	(1–4)
Number of RTX injections within 1st year after baseline, med (IQR)	4	(1–7)
Patients with at least 2 cycles, N (%)	97	(89.0)
Time duration between the first two RTX cycles (months), median (IQR)	6.1	(2.9)
Patients with less than 4 months between the first two RTX cycles, N (%)	24	(22.0)
Disease control, N (%)	97	(89.0)
Time to DC (months), median survival (SD)	7.1	(0.5)
Number of RTX cycles to achieve DC, median (range)	1	(1–6)
Time to disease control according to the site
Ocular, median survival (SD)	6.3	(2.0)
Larynx, median survival (SD)	8.2	(0.7)
Skin, median survival (SD)	3.7	(1.1)
Buccal, median survival (SD)	5.7	(0.6)
Genital, median survival (SD)	5.7	(1.4)
Anal, median survival (SD)	3.5	(0.2)
CR, N (%)	93	(85.3)
Time to CR (months), median survival (SD)	12.2	(0.4)
Number of cycles to achieve CR, median (range)	2	(1–7)
Number of injections to achieve CR, median (range)	4	(2–14)
Patients in CR after one cycle, N (%)	29	(26.6)
Patients in CR after one or two cycles, N (%)	67	(61.5)
CR achieved within 1st year after baseline, N (%)	49	(45.0)
Time duration in CR (months), median (range)	28.9	(0.7–110.4)
CR off RTX, N (%)	64	(58.7)
Time duration in CR off RTX (months), median (range)	24.9	(0.5-101.3)
CR off RTX with minimal therapy, N (%)	24	(22.0)
Time duration in CR off RTX with minimal therapy (months), median (range)	21.1	(2.4–70-9)
CR off RTX with more than minimal therapy, N (%)	40	(36.7)
Time duration in CR off RTX with more than minimal therapy (months), median (range)	21.7	(0.5–101.3)
CR on RTX, N (%)	29	(26.6)
Time duration in CR on RTX (months), median (range)	11.7	(0.7–78.9)
Relapses after CR		
First relapse^¶^, N (%)	36	(38.7)
MMPDAI at relapse^†^, median (range)	2.2	(0.0–15.0)
Number of sites involved^†^, median (range)	1	(1–3)
Ocular^†^, N (%)	16	(44.4)
Laryngeal^†^, N (%)	7	(19.4)
Buccal^†^, N (%)	11	(30.6)
Skin^†^, N (%)	9	(25.0)
Genital^†^, N (%)	1	(2.8)
CR after relapse treatment^†^, N (%)	33	(91.7)
Number of RTX cycles to achieve CR^†^, median (range)	1	0-4
Time to CR^†^, median (range)	5.6	(3.0–37.8)
Second relapse^¶^, N (%)	9	(9.7)
≥2 relapses^¶^, N (%)	1	(1.1)
Partial response, N (%)	11	(10.1)
No response, N (%)	5	(4.6)
Concomitant therapies at last follow-up, N (%)	106	(97.2)
DDS and/or sulfasalazine, N (%)	99	(90.8)
Topical corticosteroids^‡^, N (%)	1	(0.9)
Systemic corticosteroids, N (%)	1	(0.9)

MMP, mucous membrane pemphigoid; RTX, rituximab; IQR, interquartile range; DC, disease control; SD, standard deviation; CR, complete remission; MMPDAI, mucous membrane pemphigoid disease activity index; DDS, dapsone. ^*^based on 93 cases who had CR; ^†^based on 36 cases who had a relapse; ^‡^cutaneous application of more than 10g/d of high potent topical corticosteroids. ¶ corresponds to the definition of * based on 93 cases in CR.

**Figure 3:**
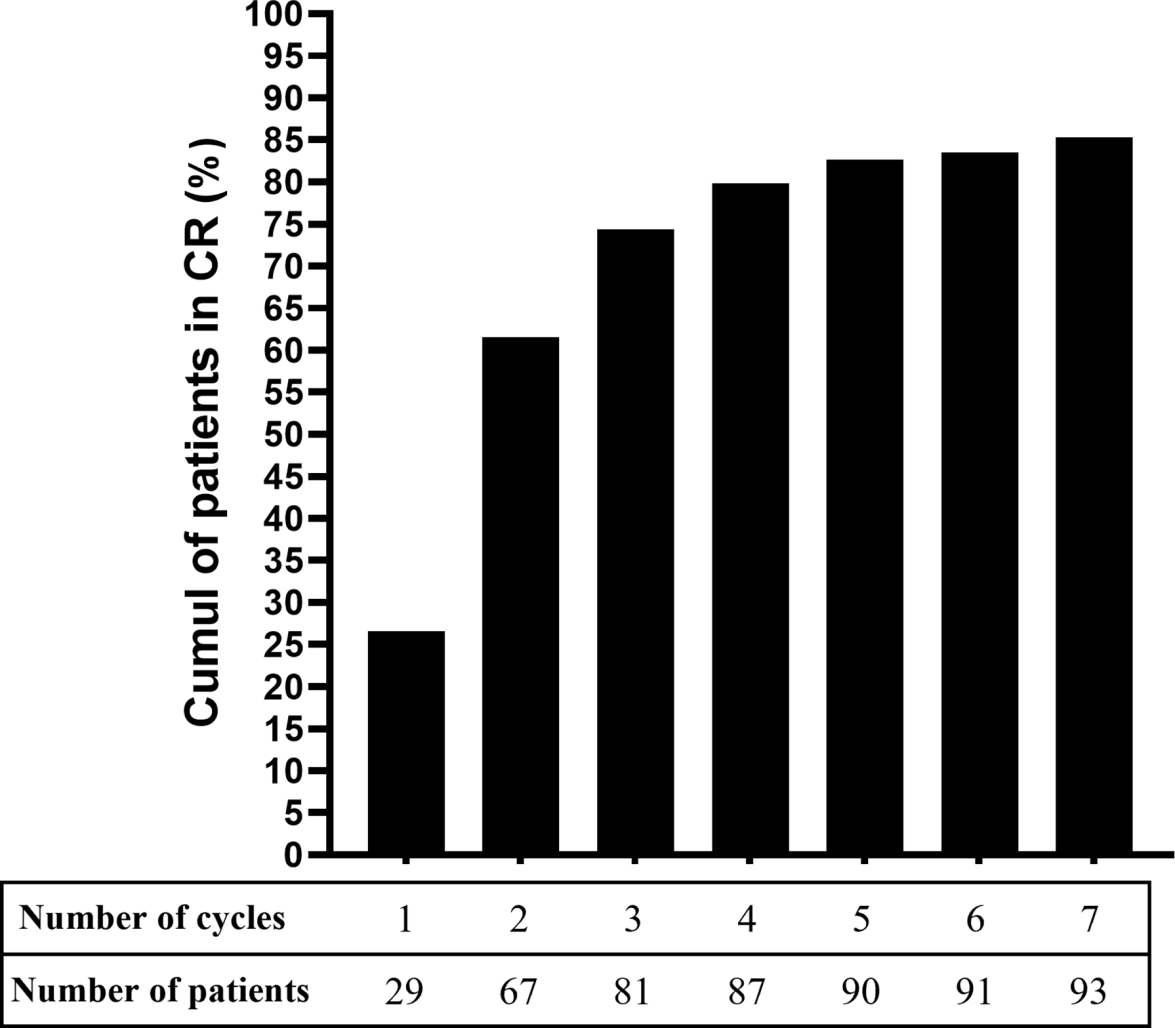
Cumulative proportion of patients with mucous membrane pemphigoid that achieved complete remission with rituximab.

After baseline, 97 patients (89.0%) achieved DC with a median survival time of 7.1 months after one RTX cycle (median) (**Table 3**). The site with the longest time taken to achieve DC was the larynx/trachea, with a median survival time of 8.2 months. Ninety-three patients (85.3%) achieved CR with a median survival time of 12.2 months after two RTX cycles (median). The number of RTX cycles to achieve CR ranged from one to seven cycles and only 29 patients (26.6%) were in CR after a single cycle (**Table 3**). Repeating the cycles according to the schedule resulted in an increase of the cumulative proportion of patients having achieved CR for the first time (**Figure 3**). This cumulative proportion reached 61.5% for those having undergone one to two cycles and 74.3% for those having undergone one to three cycles (**Figure 3**). From baseline, 49 patients (45%) achieved CR within a year. In the 93 patients that achieved CR, the median time duration in CR at the end of the follow-up was 28.9 months. During the follow-up, 64 of the 109 patients (58.7%) achieved CR off RTX, which lasted for a median time of 24.9 months; 24 patients (22.0%) achieved CR off RTX with minimal therapy, which lasted for a median time of 21.1 months; 40 patients (36.7%) achieved CR off RTX but still received higher doses of immunomodulatory drugs than those receiving minimal therapy (see methods for doses), for a median time of 21.7 months. Twenty-nine of the 109 patients (26.6%) did not achieve CR off RTX at the last follow-up and were still in CR on RTX.

After CR, 36 of the 93 patients (38.7%) experienced at least one relapse and nine (9.7%) had at least two relapses (**Table 3**). The first relapse occurred 0.8 to 110.4 months after CR and involved a median number of one site (range: 1–3 sites) with a median MMPDAI score of 2.25, which was lower than the baseline score. The most frequently involved mucosal site during relapses was the conjunctiva (44.0%). After this first relapse, CR was achieved again in 33 patients (91.7%) in 3 to 37.8 months after one cycle of RTX (median); the remaining three patients had insufficient follow-up after relapse to evaluate their response to the treatment.

At the last follow-up, 97.2% of patients underwent immunomodulatory concomitant therapies, notably dapsone or sulfasalazine (90.8%) (**Table 3**). None of the patients in CR were administered concomitant conventional ISAs and only one patient was still receiving systemic corticosteroids (0.9%). At the last follow-up, 16 patients (14.7%) did not achieve CR, 11 (10.1%) only achieved partial response, and five (4.6%) had no response to RTX. In the 16 patients without CR, a median of one site was still involved (range: 1–3) at the last follow-up and mainly comprised conjunctival (50.0%) and laryngeal mucous membranes (31.1%). RTX failure was concluded in five patients (4.6%) after five RTX cycles in median, corresponding to four of the 11 patients in partial response PR and one of the five non-responders; the four remaining cases of non-responders were not yet considered RTX failure at the last follow-up because of their short follow-up period after baseline. The five patients with RTX failure then underwent alternative therapies (IVIG, cyclophosphamide, anti-TNFα); there was no disease improvement in four patients. One of them achieved complete remission after undergoing combination therapy with anti-TNFα and IVIG.

### Adverse Events During Follow-Up After Baseline

During follow-up, 51 of the 109 patients (46.8%) had biological or clinical adverse events, of whom 23 patients (21.1%) had severe adverse events according to CTCAE grading (**Table 4**). Biological adverse events were reported in 29 patients (26.6%) and comprised lymphopenia in 28 patients (25.7%) and severe neutropenia in one patient that led to RTX cessation after the first cycle. Nevertheless, 24 of the 28 patients (84.7%) with lymphopenia already had lymphopenia before RTX therapy after having received cyclophosphamide, and none of them had a lymphocyte blood count <0.5 G/L.

**Table 4:** Adverse events during follow-up.

	All N = 109		Incidence per 100 person-year
Adverse events, N (%)	51	(46.8)	10.51
Severe adverse events, N (%)	23	(21.1)	4.74
Biological adverse events, N (%)	29	(26.6)	5.98
Neutropenia, N (%)	1	(0.9)	0.21
Lymphopenia, N (%)	28	(25.7)	5.77
Preexisting before RTX, N (%)	24	(22.0)	*na*
Clinical adverse events, N (%)	34	(31.2)	7.01
Number of cycles before adverse events, median (range)	2	(1-7)	0.41
Infectious adverse events, N (%)	20	(18.3)	4.12
Bacterial pneumopathy, N (%)	12	(11.0)	2.47
COVID-19 infection, N (%)	3	(2.7)	3.16
Flue, N (%)	1	(0.9)	0.21
Bacterial cellulitis, N (%)	1	(0.9)	0.21
Tuberculosis, N (%)	1	(0.9)	0.21
Bacterial osteitis, N (%)	1	(0.9)	0.21
Bacterial urinary tract infection, N (%)	4	(3.7)	0.82
Severe bacterial septicemia, N (%)	3	(2.8)	0.62
Candida septicemia, N (%)	1	(0.9)	0.21
Non-infectious adverse events, N (%)	12	(11.0)	2.47
Infusion reaction, N (%)	5	(4.6)	1.03
Cardiac arrythmia, N (%)	1	(0.9)	0.21
Heart failure, N (%)	1	(0.9)	0.21
NASH, N (%)	1	(0.9)	0.21
Cancer, N (%)	6	(5.5)	1.24
Death, N (%)	7	(6.4)	*na*
Age at death (years), median (range)	79.9	(41.5-91.2)	*na*
Time duration between last RTX and death, median (months), median (range)	13.2	(1.2-93.6)	*na*
Imputable to RTX, N (%)	4	(3.7)	*na*

RTX, rituximab; NASH, non-alcoholic steatosis hepatitis; na, not acquired.Number (N) and percentage (%) of patients who had adverse events.

Clinical adverse events were reported in 31.2% of the 109 patients with MMP and occurred after a median number of two cycles of RTX (range: 1–7 cycles). Most of those comprised infectious events (18.3%) with a broad spectrum of reported infections. Notably, 12 of the 109 (11.0%) patients had infectious pneumonitis, four (3.7%) had bacterial urinary tract infections, and four (3.7%) had severe bacterial or candida septicemia. Three patients (2.7%) had COVID-19 infections before COVID-19 vaccination was available. Notably, nine of the 12 patients who developed pneumonia had laryngeal involvement at baseline. The incidence for infectious diseases was 4.1 per 100 person-year. The incidences for each infectious cause were below 1.0 per 100 person-year; however, for COVID-19 infection and bacterial pneumonitis, the incidences were 3.16 and 2.47 per 100 person-year, respectively. Non-infectious adverse events occurred in 11.0% of patients; 4.6% had mild infusion reaction. Six patients (5.5%) had cancer (two breast adenocarcinomas, one of which was a local relapse from a cancer previously in remission, one prostatic adenocarcinoma, one bronchopulmonary carcinoma, and two skin carcinomas) at a median age of 74.1 years (range: 54.6–84.3 years). Except for patients with skin carcinomas, all patients with cancer adverse events had received cyclophosphamide before RTX. None of the patients who had cancer had antibodies to laminine-332.

Seven patients (6.4%) with a median age of 79.9 years died during the follow-up. Deaths occurred at a median time of 13.2 months after the last RTX infusion. Three deaths were not considered as related to RTX (one from a cerebral vascular stroke in an 83-year-old patient, one from a bronchopulmonary cancer that occurred 8 years after the last RTX infusion, and one from a ruptured aneurysm). Four deaths (3.7%) were possibly related to RTX (one from stage IV breast adenocarcinoma, one with severe bacterial pneumonia complicating an MMP-related tracheal stenosis, and two from unknown causes).

### Factors Associated With Response After RTX Therapy in Patients With MMP

First, as follow-up time durations varied among cases, we aimed at identifying factors significantly associated with CR by comparing patients with or without CR at the 8-month follow-up (**Table 5**). Univariate analyses included 101 patients with MMP of whom 27 achieved CR and 74 did not achieve CR 8 months after baseline (**Table 5**). Patients in CR at the 8-month follow-up had received significantly less RTX cycles/injections during these first months, which was in line with treatment schedule described in the methods section. Some parameters recorded had *P*-values <0.2 (age at diagnosis, MMP with anti-type VII collagen reactivity, having received or being refractory to conventional ISAs, time duration between diagnosis and baseline, ocular involvement at baseline, activity MMPDAI score at baseline, initiation or resumption of disulone or salazopyrine after baseline) but none of them demonstrated *P*-values <0.05 in univariate analysis (**Table 5**). The backward stepwise logistic-regression multivariate analyses retained the time between the first symptoms and baseline (OR, 0.986; [95% CI 0.973–0.998]; *P* = 0.0265), the activity MMPDAI score at baseline (OR 0.944; [95% CI 0.890–1.00]; *P* = 0.0495) and being refractory to conventional ISAs (OR 0.299; [95% CI 0.105–0.849]; *P* = 0.0495) as factors associated with the absence of CR at the 8-month follow-up. For this logistic-regression model, R-squared was 0.117.

**Table 5:** Univariate analysis: factors associated with complete remission at 8-month follow-up in 101 patients with MMP.

Variables	All* N = 101		No CRN = 74		CRN = 27		P-value**	
Female gender, N (%)	52	(51.5)	36	(48.7)	16	(59.3)		0.3450
Age at diagnosis (years), median (IQR)	69.7	(21.5)	66.8	(26.4)	70.9	(18.6)		0.0684
Time duration (months) between symptoms and diagnosis, median (IQR)	15.9	(33.4)	15.4	(38.0)	15.9	(21.8)		0.7443
Immune deposits at BMZ in DIF IgG, N (%)^†^ IgA, N (%)^†^ IgG and IgA, N (%)^†^ Exclusive IgA, N (%)^†^ C3, N (%)^†^	73 37 29 8 72	(77.7) (39.4) (30.9) (8.5) (72.6)	53 28 22 6 53	(77.9) (41.2) (32.4) (8.8) (77.9)	20 9 7 2 19	(76.9) (34.6) (26.9) (7.7) (73.3)		>0.9999 0.7290 0.7947 >0.9999 0.8212
Anti-BP180 antibodies, N (%)	30	(29.7)	21	(28.4)	9	(33.3)		0.8113
MMP with anti-type VII collagen reactivity, N (%)	12	(11.9)	11	(14.9)	1	(3.7)		0.1735
Mucosal involvement only at diagnosis, N (%)	50	(49.5)	38	(51.4)	12	(44.4)		0.5389
Ocular monosite MMP, N (%)	17	(16.8)	11	(14.9)	6	(22.2)		0.5639
Severe disease at diagnosis, N (%)	97	(96.0)	71	(95.9)	26	(96.3)		>0.9999
Therapeutic lines before baseline								
Immunomodulatory drugs^¶^, N (%)	81	(80.2)	58	(78.4)	23	(85.2)		0.6309
DDS/salazopyrine, N (%)	68	(67.3)	48	(64.9)	20	(74.1)		0.5248
ISAs, N (%)	67	(66.4)	53	(71.6)	14	(51.9)		0.1041
Cyclophosphamide, N (%)	52	(51.5)	39	(52.7)	13	(48.1)		0.6582
Previously had a period of CR before baseline, N (%)	9	(8.9)	8	(10.8)	1	(3.7)		>0.4381
Age at baseline (years), median (IQR)	70.5	(19.0)	70.2	(25.4)	71.0	(18.0)		0.1417
Time duration between first symptoms and baseline (months), median (IQR)	34.2	(63.5)	41.4	(83.3)	32.1	(35.8)		0.1258
Time duration between diagnosis and baseline (months), median (IQR)	7.3	(13.3)	7.1	(18.1)	7.5	(14.3)		0.3203
RTX indication								
Refractory/contraindication to immunomodulatory drugs^¶^, N (%)	78	(77.2)	57	(77.0)	21	(77.8)		>0.9999
Refractory to ISAs, N (%)	66	(65.3)	52	(70.3)	14	(51.9)		0.0852
Time duration of ISAs treatment (months), median (IQR)	4.0	(6.0)	4.0	(5.0)	6.0	(9.0)		0.4846
Contraindication to conventional ISA, N (%)	23	(22.8)	16	(21.6)	7	(25.9)		0.8484
Baseline Involvement								
Number of sites involved, median (IQR)	2.0	(2.0)	2.0	(2.0)	2.0	(2.0)		0.3843
Ocular, N (%)	52	(51.5)	41	(55.4)	11	(40.7)		0.1919
Laryngeal, N(%)	42	(41.6)	33	(44.6)	9	(33.3)		0.4293
Buccal, N (%)	63	(62.4)	46	(62.2)	17	(63.0)		0.9414
Skin, N (%)	29	(28.7)	22	(29.7)	7	(25.9)		0.8982
Genital, N(%)	12	(11.9)	9	(12.2)	3	(11.1)		>0.9999
Anal, N (%)	12	(11.9)	10	(13.5)	2	(7.4)		0.5069
Esophagus, N (%)	7	(6.9)	6	(8.1)	1	(3.7)		0.6713
≥1 site involved, N (%)	68	(67.3)	51	(68.9)	17	(63.0)		0.5722
≥2 sites involved, N (%)	43	(42.6)	33	(44.6)	10	(37.0)		0.4966
Baseline activity MMPDAI score, median (IQR)	11.0	(13.7)	11.7	(11.2)	7.0	(16.7)		0.1478
Concomitant treatments at baseline								
Immunomodulatory drugs^¶^, N (%)	78	(77.2)	56	(75.7)	22	(81.5)		0.7261
DDS and/or salazopyrine, N (%)	68	(67.3)	48	(64.9)	20	(74.1)		0.5248
Systemic corticosteroid, N(%)	4	(4.0)	4	(5.4)	0	(0.0)		>0.5713
Topical corticosteroids^‡^, N (%)	16	(15.9)	10	(13.5)	6	(22.2)		0.4497
Number of RTX cycles in 8 months, median (IQR)	2	(1.0)	2	(0.0)	2.0	(1.0)		0.0118
Number of RTX injections in 8 months, median (IQR)	4	(2.0)	4	(1.0)	3	(1.7)		0.0001
≥ 3 RTX injections, N (%)	61	(60.4)	54	(73.0)	7	(25.9)		<0.0001
Concomitant immunomodulatory drugs after baseline, N (%)	99	(98.0)	73	(98.7)	26	(96.3)		0.4651
Initiation/resumption of DDS/salazopyrine after baseline, N (%)	27	(26.7)	23	(31.1)	4	(14.8)		0.1304

CR, complete remission; IQR, interquartile range; BMZ, basement membrane zone; DIF, direct immunofluorescence; DDS, dapsone; ISAs, immunosuppressive agents; RTX, rituximab; MMPDAI, mucous membrane pemphigoid disease activity index. *Only the 101 patients with MMP and more than 8 months of follow-up after baseline were included in this analysis. **univariate comparison analyses between the CR and non-CR groups. ^†^based on 94 cases; ^¶^i.e., DDS, Sulfasalazine, doxycycline or equivalent, hydroxychloroquine, acitretin or colchicine; ^‡^cutaneous application of more than 10g/d of high potent topical corticosteroids.

**Figure 4:**
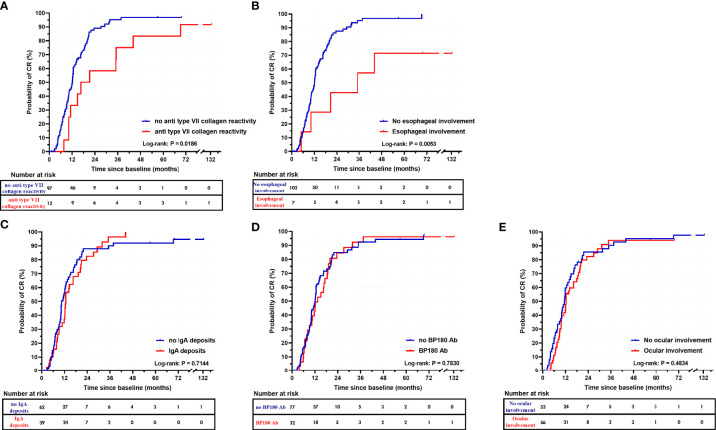
Kaplan-Meier survival curves for complete remission. Survival curves for complete remission in patients with MMP with anti-type VII collagen reactivity and MMP without anti-type VII collagen reactivity **(A)**, and depending on the presence of esophageal involvement **(B)**, IgA deposits in DIF or DIEM **(C)**, circulating anti-BP180 antibodies **(D)**, or ocular involvement **(E)**.

Second, we aimed at identifying parameters significantly associated with a longer survival time to CR. The same parameters as for the analyses at the 8-month follow-up were studied. Patients with MMP with anti-type VII collagen reactivity achieved CR in a longer time (*P* = 0.0186, median survival time: 16.6 months) in comparison with patients with MMP without anti-type VII collagen reactivity (median survival time: 12.1 months) (**Figure 4A**). Patients with an esophageal involvement also achieved CR in a longer time (*P* = 0.0053, median survival time: 35.1 months) in comparison with patients without esophageal involvement (median survival time: 12.1 months) (**Figure 4B**). The difference observed according esophageal involvement might be dependent on the “MMP with anti-type VII collagen reactivity” parameter. Indeed, patients with MMP with anti-type VII collagen reactivity had significantly (*P* = 0.026) more frequently esophageal involvement at baseline (33.3%) in comparison with other patients (3.1%) and demonstrated the longer time to achieve CR among patients with esophageal involvement. For the other parameters studied, the groups had no significant difference in the time to achieve CR. Notably, the survival analysis comparing the groups with or without IgA deposits in DIF or DIEM, circulating anti-BP180 antibodies, or ocular involvement at baseline showed no significant difference (**Figures 4C–E**). Noteworthy, as patients with MM-EBA, patients with ocular involvement had received more RTX injections during the first year (*P* = 0.0092) of follow-up and during the entire follow-up (*P* = 0.0583) than those without ocular involvement.

**Figure 5:**
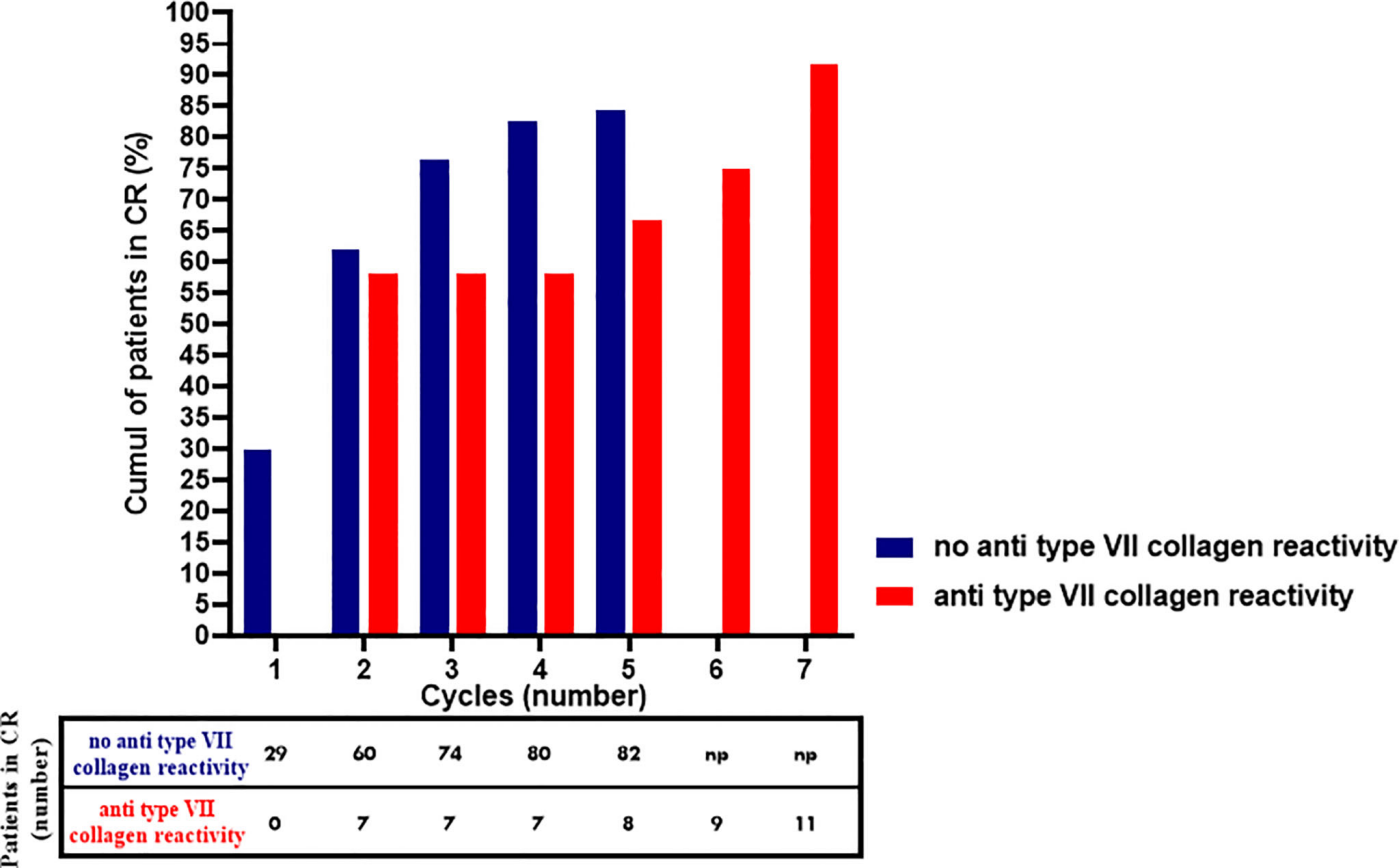
Cumulative proportion of patients with MMP with anti-type VII collagen reactivity MMP without anti-type VII collagen reactivity that achieved complete remission with rituximab. *np, no RTX cycle performed*.

At the end of the follow-up, the CR rate was not significantly different between patients with MMP with anti-type VII collagen reactivity and other patients (P > 0.9999). Nevertheless, patients with MMP with anti-type VII collagen reactivity had required significantly more RTX cycles/injections (*P* = 0.0186) than other patients with MMP to achieve CR (**Figure 5**).

### Factors Associated With Relapse in Remittent Patients With MMP After RTX

Univariate analyses aiming to identify factors associated with the relapse at the 6-month follow-up after CR included 86 patients, 68 of whom did not relapse and 13 of whom did (**Table 6**). No parameter was found significantly associated with the relapse in univariate and multivariate analyses.

**Table 6:** Univariate analysis: factors associated with relapse within 6 months after complete response in 86 patients with MMP.

Variables	All CR* N = 81		No relapse N = 68		Relapse N = 13		P-value**
Female gender, N (%)	45	(52.3)	41	(56.2)	4	(30.8)	0.1323
Age at diagnosis (years), median (IQR)	69.7	(19.8)	69.7	(19.2)	74.0	(25.2)	0.6042
Delay between symptoms and diagnosis (months), median (IQR)	14.8	(29.5)	14.6	(30.4)	16.5	(27.8)	0.6211
Immune deposits at BMZ in DIF IgG, N (%)^†^ IgA, N (%)^†^ IgG and IgA, N (%)^†^ Exclusive IgA, N (%)^†^ C3, N (%)^†^	62 33 26 7 65	(7.5) (40.7) (32.1) (8.6) (76.5)	53 28 22 6 54	(77.9) (41.2) (32.4) (8.8) (79.4)	9 5 4 1 8	(69.2) (38.5) (30.8) (7.7) (61.5)	0.4909 >0.9999 >0.9999 >0.9999 0.2980
MMP with anti-type VII collagen reactivity, N (%)	10	(11.6)	8	(11.0)	2	(15.4)	0.6436
Anti-BP180 positivity, N (%)	25	(35.2)	22	(36.7)	3	(27.3)	0.5330
Mucosal involvement only at diagnosis, N (%)	42	(59.2)	37	(61.7)	5	(45.5)	0.6092
Ocular monosite MMP, N (%)	12	(14.0)	9	(12.3)	3	(27.1)	0.3805
Severe MMP, N (%)	83	(96.5)	70	(95.9)	13	(100)	>0.9999
Therapeutic lines before baseline							
Immunomodulatory drugs^¶^, N (%)	68	(79.1)	56	(76.7)	12	(92.3)	0.2847
DDS/salazopyrine, N (%)	57	(66.3)	47	(64.4)	10	(76.9)	0.5290
ISA, N (%)	57	(66.3)	46	(63.0)	11	(84.6)	0.2035
Cyclophosphamide, N (%)	44	(51.2)	34	(46.6)	10	(76.9)	0.0690
Previously experience CR before RTX, N (%)	8	(9.3)	7	(9.6)	1	(7.7)	>0.9999
Age at baseline (years), median (IQR)	70.9	(17.7)	70.9	(17.1)	74.3	(20.6)	0.5875
Time duration between first symptoms and baseline (months), median (IQR)	36.3	(60.9)	38.5	(62.2)	32.2	(38.1)	0.5466
Time duration between diagnosis and baseline (months), median (IQR)	6.8	(14.9)	6.1	(16.3)	8.5	(12.8)	0.4658
RTX indication							
Refractory/contraindication to immunomodulatory drugs, N (%)	69	(80.2)	57	(78.1)	12	(92.3)	0.1727
Refractory to ISA, N (%)	56	(65.2)	45	(61.6)	11	(84.6)	0.1286
Time duration of ISA treatment (months), median (IQR)	3.5	(6.5)	3.0	(7.0)	5.0	(3.7)	0.9616
Contraindication to conventional ISA, N (%)	19	(22.1)	18	(24.7)	1	(7.7)	0.2810
Baseline Involvement							
Number of sites involved, median (IQR)	2.0	(2.0)	2.0	(2.0)	2.0	(3.0)	0.9056
Ocular, N (%)	42	(48.8)	35	(47.9)	7	(53.8)	0.9276
Laryngeal, N(%)	37	(43.0)	32	(43.8)	5	(38.5)	0.9549
Buccal, N (%)	55	(64.0)	46	(63.0)	9	(69.2)	0.7623
Skin, N(%)	26	(30.2)	22	(30.1)	4	(30.8)	>0.9999
Genital, N(%)	11	(12.8)	9	(12.3)	2	(15.4)	0.6698
Anal, N (%)	9	(10.5)	8	(11.0)	1	(7.7)	>0.9999
Esophagus, N(%)	5	(5.8)	4	(5.5)	1	(7.7)	0.5687
≥1 site involved, N (%)	61	(70.9)	52	(71.2)	9	(69.2)	>0.9999
≥2 sites involved, N (%)	36	(41.9)	31	(42.5)	5	(38.5)	>0.9999
Baseline activity MMPDAI score, median (IQR)	11.0	(13.0)	11.5	(13.2)	10.5	(12.5)	0.6641
Concomitant treatments at baseline							
Immunomodulatory drugs^¶^, N (%)	64	(74.4)	53	(72.6)	11	(84.6)	0.5005
DDS and/or salazopyrine, N(%)	55	(64.0)	45	(61.7)	10	(76.9)	0.3608
Systemic corticosteroid, N (%)	2	(2.3)	2	(2.7)	0	(0)	>0.9999
Topical corticosteroids^‡^, N(%)	14	(16.3)	12	(16.4)	2	(15.4)	>0.9999
Less than 4 months between the two first cycles, N (%)	16	(20.8)	13	(20.0)	3	(25.0)	0.7055
Time to CR (months), median (IQR)	11.7	(9.0)	11.8	(9.2)	11.4	(5.8)	0.6863
Number of RTX cycles to achieve CR, median (IQR)	2.0	(2.0)	2.0	(2.0)	2.0	(1.2)	0.6586
Number of RTX injections to achieve CR, median (IQR)	4.0	(4.0)	4.0	(3.2)	4.0	(3.2)	0.6823
Concomitant immunomodulatory drugs after baseline, N (%)	84	(97.7)	71	(97.3)	13	(100.0)	>0.9999
Initiation/resumption of DDS/salazopyrine after baseline, N (%)	25	(29.1)	22	(30.1)	3	(23.1)	0.7480

CR, complete remission; IQR, interquartile range; BMZ, basement membrane zone; DIF, direct immunofluorescence; Ab, antibody; MMP, mucous membrane pemphigoid; DDS, dapsone; ISA, immunosuppressive agents; RTX, rituximab; MMPDAI, mucous membrane pemphigoid disease activity index. *Only the 86 patients with MMP and more than 6 months of follow-up after complete response were included in this analysis, **univariate comparison analyses between the CR and non-CR groups. ^†^based on 81 cases; ^¶^ i.e., DDS, Sulfasalazine, doxycycline or equivalent, hydroxychloroquine, acitretine or colchicine; ^‡^cutaneous application of more than 10g/d of high potent topical corticosteroids.

## Discussion

RTX is a murine-human chimeric monoclonal antibody directed against CD20, a cell surface marker expressed by B cells after the late pre B-cell stage (except plasma cells), and is responsible for prolonged B-cell depletion followed by a 6-month delayed recovery period (19, 20). Following clinical trials, meta-analyses, and a prospective multicenter randomized trial (21–24), RTX is now recommended in first line therapy for moderate to severe pemphigus in combination with oral corticosteroids (25, 26). RTX has also been used off-label, alone or in combination therapies, with success in a wide range of other refractory AIBDs, including MMP (10, 11, 27, 28). Herein, we reported a large monocentric retrospective real-life series of patients with MMP treated with RTX without conventional ISAs. We established that RTX in association with immunomodulatory drugs is an effective therapeutic option in severe and/or refractory MMP, achieving a cumulative CR rate of 85.3% after one or multiple cycles. Our series provides a large contribution to the field as 109 patients were included with a long-term follow-up of 51.4 months (median). In comparison, the largest study to date on patients with MMP treated with biologics was a recent systematic review from Lytvyn et al. (11). This review involved 63 studies and included 331 patients, 112 of whom were treated with RTX, and some of them concomitantly received conventional ISAs (10, 27).

### Limitations

Since MMP is rare, there is a limitation regarding the number of patients that can be recruited in studies. Our study was retrospective, covering a period of 10 years that corresponded to a change in our therapeutic management of patients with MMP since concomitant treatments with RTX (conventional ISAs) were stopped in the center to avoid side effects. This study was monocentric, performed on a cohort of patients followed up in a French referral center for AIBD, that ensures a homogenous management by a multidisciplinary team regarding systematic evaluation, diagnoses, and therapeutic schedule for RTX and concomitant therapies. Despite screening 121 patients for inclusion, this monocentric setting might have led to a loss of statistical power. Since patients were referred from other centers in France or nearby countries, this might have induced loss of sight when patients wished to continue their follow-up closer to their home, and might have therefore caused the exclusion of some cases or shortened follow-up duration. Nevertheless, 109 patients were included with a median follow-up duration >4 years. Since non-responders with severe MMP might have received a second RTX cycle 3 months after baseline on an individual basis (see Methods), this might have resulted in the administration of different RTX regimens according to disease severity. This indication bias might have reduced our chance to identify clinical factors influencing the response to RTX as severely affected patients underwent more RTX injections during the first year of follow-up.

### Comparison of the Study Population With Previous Literature

Our study population had similar epidemiologic characteristics as those in previous studies; there was a predominance of females (11, 29–31) as well as diseases lasting for several years before RTX therapy since MMP diagnosis had been delayed for several months to years (28, 30, 32). The median age at MMP diagnosis (69.7 years) was in line with average values reported in other studies (3, 9, 31), but was higher than that in the systematic review by Lytvyn et al. (11). At the time of diagnosis, the most common sites involved in our study in decreasing order of frequency were nasopharyngeal, oral, ocular, laryngeal, genital, and anal MM, which was comparable with previous findings (1, 30, 31, 33), apart from a higher proportion of nasopharyngeal involvement. In comparison with the study by Lytvyn et al., our series had less frequent ocular involvement (59.6% vs. 70.1%) but more frequent laryngo-tracheal (49.5% vs. 23.9%) and buccal involvement (65.1 vs. 39%), in line with previous publications by our multidisciplinary team (34). These differences might be a consequence of the large amount of studies involving ocular monosite MMP in the review by Lytvyn et al. and from center specificities in the multidisciplinary evaluation; notably, patients with MMP were systematically examined by a stomatologist, an ophthalmologist, and an otorhinolaryngologist in our referral center, which might explain higher rates of ENT involvement considering that the latter might be asymptomatic (34, 35). The rate of skin involvement was similar to that in previous studies (3). The proportion of patients with MMP having symptomatic esophageal disease was higher in this series (8.3%) than in our previous retrospective study that included 477 patients with MMP (36).

The population study demonstrated similar severity in comparison with other retrospective studies including patients with MMP, considering the proportion of severe disease (95.4%), patients with ≥3 sites (67.0%) at diagnosis, and patients with multisite involvement (64.2%) at baseline (9, 10, 27, 30). Regarding therapeutics prior to baseline, fewer patients had been treated with systemic corticosteroids in our study (11.0%) in comparison with the pooled population from the systematic review (11). This discrepancy resulted from local management guidelines aimed at preventing long-term use of corticosteroids and its inherent complications, according our previous therapeutic experience with MMP (9, 10). A much larger proportion of our patients had experienced failure with immunomodulatory therapies (80.7%) and conventional ISAs (66.1%) in comparison with the patients included in the systematic review (35.0% and 46.5%, respectively); however, these therapies had reduced the number of sites affected at baseline (median: 2 sites) in comparison with diagnosis (median value: 3 sites)

Interestingly, in comparison with the 25.1% of patients who received concomitant ISAs with RTX therapy as reported in the systematic review (11), only 4.6% of our patients received concomitant systemic corticosteroids and none received other ISAs. Contrastingly, a higher proportion of patients received concomitant immunomodulatory drugs (78.0% vs. 15.1%). Thus, this series allowed us to better evaluate RTX efficacy in combination with immunomodulatory drugs.

### Outcomes of Patients With MMP Treated With RTX and Factors Associated With the Outcomes

DC is rarely analyzed in studies on MMP. The percentage of patients that achieved DC in our series (89%) was lower than the DC rate of 100% reported in a previous study involving patients receiving RTX and conventional therapies, including immunosuppressants (27). Nevertheless, the DC rate in our study was higher compared to those reported in studies by Lamberts et al. and Rashid et al. (67.9% and 81%, respectively) (28, 33). The median survival time to DC (7.1 months) in our series was shorter than the average time to DC (10.2 months) reported by Maley et al., but was longer than the 14.5 weeks reported by Lamberts et al. (28). We used Kaplan-Meier curves to estimate the median survival in order to take into account the disparity of follow-up times and censures across patients; this might explain the difference in median time observed in those studies. Regarding DC according to sites, we found that laryngeal involvement had a longer median survival time than the other sites involved. Moreover, the larynx was still affected in 31% of patients without CR at the last follow-up. These results support other studies highlighting that laryngeal involvement may be more refractory to RTX (37). The conjunctivae was the site with the second longest median survival time to DC (6.3 months), which was close to the medial survival times of buccal and genital involvement (5.7 months). Thus, ocular lesions did not take longer to heal than other mucosal sites such as buccal or genital mucous membrane, as suggested in previous studies (9, 38, 39).

RTX regimen and concomitant therapies differ in retrospective studies reporting RTX efficacy in patients with MMP, making it difficult to compare the resolution outcomes (10, 11, 27, 28). Only 4.6% of patients were non-responders to RTX and 95.4% were responders (85.3% with CR; 10.1% with partial response) in our series. These results are similar to those of a previous study at our center, which reported 92% of responder patients after one or two RTX cycles (10). Nevertheless, the RTX regimen differed between the two studies and the median number of RTX cycles received was higher in the current one. The definition of CR also differed between the two studies and required to have complete healing and no new lesions for 2 months in the current one. Besides, a second cycle of RTX was systematically proposed 4 to 6 months after baseline in non-CR patients to achieve CR and 6 months after baseline in CR patients to consolidate the remission in the current series. These parameters might explain why the CR rate after a unique cycle was 68% in the previous study and only 26.6% in the current one. The proportion of responders (95.4%) is notably much higher than that reported in the study by Lamberts et al. (57.1%) which applied a similar regimen with RTX injections at baseline, 6 and 12 months, but with lower dosage (500 mg) of RTX after baseline (28). The number, frequency, and dosage of RTX injections after baseline might thus influence the response in patients with MMP. However, we could not test this hypothesis in this study due to the indication bias that was responsible for a more aggressive regimen in some initial non-responders.

Overall, the cumulative CR rate we achieved (85.3%) was similar to that reported in our previous study (10). CR was achieved after two RTX cycles (median), corresponding to four RTX infusions (median), which confirmed our previous observations regarding the benefit of completing at least two RTX cycles in patients with MMP (10). In patients who were non-responders or partial responders after the first two cycles, repeated RTX cycles increased the CR rate subsequently (**Figure 3**). The cumulative proportion of CR (85.3%) was higher than that reported in the systematic review by Lytvyn et al. (70.5%), whereas the proportion of non-responders was similar (5.4%) (11). Our population study showed similar severity in comparison with the literature, and much fewer patients received concomitant ISA, whereas the proportion of patients receiving immunomodulatory drugs was higher (11). Thus, the use of concomitant immunomodulatory drugs might have contributed to this difference in the proportion of CR between our study and the previous review (11). The median time taken to achieve CR was similar (12.2 months vs. 10.1 months) considering the differences in outcome definition and in the calculation methods; notably, the definition of CR we used required the absence of lesions for 2 months.

While it has been suggested in recent guidelines to avoid differentiating subtypes of MMP (2), we found a significant difference (*p =* 0.0186) in the median survival time to achieve CR between patients with MMP with anti-type VII collagen reactivity (16.6 months) and those with MMP without anti-type VII collagen reactivity (12.1 months), although there was no significant difference in the percentage of patients achieving DC and CR. Patients with MMP with anti-type VII collagen reactivity required more RTX cycles to achieve DC and CR, both of which took longer to achieve. This difference was not found in a study comparing MMP and EBA outcomes that included less patients (28). We believe that this finding is important as MMP with anti-type VII collagen reactivity patients seem more difficult to control in our experience. Thus, repeating RTX cycles in patients with MMP with anti-type VII collagen reactivity and waiting for progressive improvement until CR might constitute a preferred option in cases of severe or refractory disease, rather than shifting to another ISA-based therapy. Notably, all of our five patients with therapeutic failure had MMP without anti-type VII collagen reactivity and only one achieved a better outcome after RTX was replaced by a combination therapy of anti-TNFα and IVIG.

To our knowledge, only a few studies with smaller sample sizes have investigated factors that might be associated with a poorer response to RTX in patients with multisite MMP (28). As described above, statistical analyses might suffer from a lack of power considering the number of patients included and the indication bias that predisposed non-responder patients with severe disease to receive the second RTX cycle earlier and undergo more RTX cycles within the first year. These limitations possibly prevented us from identifying more factors significantly associated with CR at the 8-month follow-up in univariate analyses. None of the parameters studied demonstrated *P-*values <0.05 in univariate analyses. Notably, a higher percentage of patients that did not achieved CR at the 8-month follow-up had ocular involvement (55.4% vs. 40.7%) but the ocular involvement was not significantly associated with CR status in univariate analysis (*P* = 0.1919) and multivariate analysis. As stated above, patients with ocular involvement had received significantly more RTX injections during the first year of the follow-up which might have biased the results. The median activity score of MMPDAI differed according to CR status at 8 months (11.7 vs. 7.0) but was not significantly associated with CR status after 8 months of follow-up in univariate analysis (*p* = 0.1478). As stated in the 2015 consensus statement, the MMPDAI score is a validated score that is suitable for use by dermatologists and multidisciplinary teams for milder forms of MMP (12). As other scores assessing monosite or multisite MMP activity, MMPDAI does not consider laryngo-tracheal lesions. As laryngeal involvement was present in 39.4% of our patients at baseline and considering that this location demonstrated the longest time taken to achieve DC, a score integrating laryngeal activity might have provided significant information. Scores other than MMPDAI do exist, notably for specific site scoring, and some adapted scores were used in previous studies (3, 9, 40); however, we favored the use of the validated scoring system. Nevertheless, in multivariate analyses the time between the first symptoms and baseline, the MMPDAI score at baseline and being refractory to conventional ISAs were significantly associated with the absence of CR at 8-months follow-up. Considering the small sample size and the R-squared value, these results should be interpreted with caution and should be confirmed in unbiased cohorts.

Whereas some studies found IgA-dominant cases to have poorer outcomes (28), our analyses regarding IgA deposits did not find this parameter to be associated with CR status and time to CR did not differ significantly in patients with IgA deposits.

The relapse rate in this study was similar to those reported in the literature (10, 27). As described in shorter series, most patients achieved CR again (91.7%) after relapse in a short time with a median number of one RTX cycle.

After CR, RTX was carefully stopped, following which immunomodulatory drugs were progressively tapered up to the doses defined as minimal therapy in the consensus conference. The therapeutic schedule in our center did not include the cessation of immunomodulatory drugs to prevent relapses. The RTX schedule, which comprised a consolidation injection after CR, allowed 58.7% of the 109 patients to achieve CR off RTX (68.8% of patients in CR) and 22.0% of patients to achieve CR off RTX with minimal therapy (25.8% of patients in CR). These results highlight that our RTX regimen and therapeutic schedule allowed a majority of severe and resistant patients to pass a milestone and to be subsequently controlled by non-immunosuppressive treatment with a better tolerance profile than conventional ISAs or corticosteroids. Comparing the treatment schedule with other published studies is delicate. Notably, RTX reinjection at 6 and 12 months has been performed in some of these studies (28, 33) but it is difficult to ascertain that these injections were administered to CR patients, whereas our protocol comprised RTX cycles every 6 months until CR followed by a unique infusion 6 months later to consolidate the CR. At the last follow-up, 36.8% of patients achieved CR off RTX with doses of immunomodulatory drugs superior to those defined as minimal therapy (43.0% of patients in CR) for a time duration up to 101.3 months, showing that some patients required long-term administration of high doses of these drugs to prevent relapse. Finally, 26.6% of patients with MMP remained in CR on RTX (31.2% of patients in CR). The median time spent by these patients in CR on RTX at the last follow-up was 11.7 months, but it ranged from 0.7 to 78.9 months; this highlights that some of these patients received RTX injections intermittently on a long-term basis to prevent relapse in severe or difficult-to-control cases. In these specific patients, chronic reinjection of RTX over several years to maintain CR or PR may raise concerns about its financial sustainability, and a careful assessment of long-term tolerance is required with respect to this specific population.

### RTX Tolerance: Adverse Events

Our series reported a high rate of adverse events (46.8%), but only 21.1% of patients had severe adverse events. Notably, a high percentage of patients receiving RTX had lymphopenia without disturbance in other hematopoietic cell lineages, which mainly occurred after the first cycle of RTX in patients previously having received cyclophosphamide. Lymphopenia and secondary malignancies are well-known complications of cyclophosphamide use (9, 41), whereas they are uncommon adverse events associated with RTX use in AIBD (42). Previous therapies received by aged population of patients with MMP as well as long term follow-up might have thus contributed to the reported adverse event rates. The 5.5% rate of incident cancer in our current series was similar to that reported in other large series (30). Moreover, a higher cancer prevalence (11.7%) had been reported previously in a French multicenter retrospective cohort of patients with MMP and was found to not differ from the general population within the same age range in France (43). In concordance with this study (43), a higher incidence of cancer was not found in patients having anti-laminin 332 antibodies in our studies, whereas it was suggested in other cohorts of patients with MMP (30, 31, 44). Non-infectious and infectious adverse events reported in our cohort included those commonly reported with RTX, notably in other AIBDs such as pemphigus (42). The percentage of patients with infectious pneumonia adverse events was higher than in other cohorts (11, 28). The latter might have been increased by the COVID-19 pandemic that occurred in 2019; in this regard, a study in France previously reported that patients with AIBD receiving RTX had a 5-fold higher incidence of COVID-19 infection than patients who did not receive RTX during the first COVID-19 wave (45). Patients with AIBD with COVID-19 also demonstrated a 5.9-fold higher risk of dying (45), but no patient in this series had fatal issues. Finally, the incidences of infectious adverse events and pneumonia were lower than those reported in a study that explored their incidence according to age in a series of patients with auto-immune diseases receiving RTX (46).

In conclusion, RTX associated with immunomodulatory drugs is an effective and safe treatment in refractory and severe MMP, achieving DC in 89.0% of cases in 7.2 months and CR in 85.3% of cases after two cycles of RTX in 12.2 months. Similar values of high efficacy have been obtained with RTX in other AIBD such as pemphigus (24). RTX therapy in patients with MMP might be more effective than in those with bullous pemphigoid (47, 48). Although 38.7% of patients experienced relapse, CR off RTX was achieved in 68.8% of patients that had CR, whereas CR with minimal therapy was only achieved in 31.2% of them. Thus, RTX allowed patients with MMP in therapeutic impasse to pass a milestone. Our findings indicate that the continuation of immunomodulatory drugs may be mandatory to maintain patients in a state of long-term remission. Prospective comparative studies are required to confirm these results and define the position of RTX in the therapeutic armamentarium for MMP. Important information might notably be obtained from an ongoing phase 3 clinical trial comparing the safety and effectiveness of RTX vs. oral cyclophosphamide in MMP (NCT 03295383).

## Data Availability Statement

The raw data supporting the conclusions of this article will be made available by the authors, without undue reservation.

## Ethics Statement

The studies involving human participants were reviewed and approved by Comité local d’éthique pour la recherche clinique des HUPSSD Avicenne-Jean Verdier-René Muret, CH Avicenne, AP-HP, Bobigny, France (#CLEA-2022-236). Written informed consent to participate in this study was provided by the participants’ legal guardian/next of kin.

## Author Contributions

MA, GB, CP, and PM contributed to the conception and the design of the study. MA, CL-V, GB, LR, CZ, BM, FC, and CP handled the patients’ daily care and dermatological data collection. ISo was involved in ENT assessment and data collection. SD and EG performed ophthalmological assessment and data collection. ISi performed stomatological assessment and data collection. FM and SG-M performed the serum immunological analyses. MH, NL, and CP performed direct immunoelectron microscopy. GB, MA, and BM reviewed the charts of the patients and organized the databases. GB and JS performed the statistical analysis and figures. GB wrote the first draft of the manuscript. All authors contributed to manuscript revision and have read and approved the submitted version.

## Conflict of Interest

GB, MA, CL-V, FC, CP-P, and PM were investigators in the “Ritux 3” study (NCT00784589) and the “Pemphix” trial (NCT02383589) conducted by Roche Laboratories. MA, CL-V, FC, CP-P, and PM are investigators in “RTX-MMP” study (NCT03295383).

The remaining authors declare that the research was conducted in the absence of any commercial or financial relationships that could be construed as a potential conflict of interest.

## Publisher’s Note

All claims expressed in this article are solely those of the authors and do not necessarily represent those of their affiliated organizations, or those of the publisher, the editors and the reviewers. Any product that may be evaluated in this article, or claim that may be made by its manufacturer, is not guaranteed or endorsed by the publisher.

## Abbreviations

AIBD, auto-immune blistering disease; BP180, bullous pemphigoid 180 kDa antigen; BP180-NC16A, non-collagenous 16 A region of BP180; BP230, bullous pemphigoid 230 kDa antigen; CR, complete remission; DC, disease control; DIF, direct immunofluorescence; DIEM, direct immunoelectron microscopy; IVIG, intravenous immunoglobulins; ISA, immunosuppressants; MMP, mucous membrane pemphigoid; MMPDAI, mucous membrane pemphigoid disease activity index; RTX, rituximab.
